# 20 GHz fiber-integrated femtosecond pulse and supercontinuum generation with a resonant electro-optic frequency comb

**DOI:** 10.1063/5.0165681

**Published:** 2023

**Authors:** Pooja Sekhar, Connor Fredrick, David R. Carlson, Zachary L. Newman, Scott A. Diddams

**Affiliations:** 1 Department of Physics, University of Colorado Boulder, 440 UCB, Boulder, Colorado 80309, USA; 2 Time and Frequency Division, National Institute of Standards and Technology, 325 Broadway, Boulder, Colorado 80305, USA; 3 Octave Photonics, 325 W South Boulder Rd., Louisville, Colorado 80027, USA; 4 Department of Electrical, Computer and Energy Engineering, University of Colorado Boulder, Colorado 80309, USA

## Abstract

Frequency combs with mode spacing of 10–20 GHz are critical for increasingly important applications such as astronomical spectrograph calibration, high-speed dual-comb spectroscopy, and low-noise microwave generation. While electro-optic modulators and microresonators can provide narrowband comb sources at this repetition rate, a significant remaining challenge is a means to produce pulses with sufficient peak power to initiate nonlinear supercontinuum generation spanning hundreds of terahertz (THz) as required for self-referencing. Here, we provide a simple, robust, and universal solution to this problem using off-the-shelf polarization-maintaining amplification and nonlinear fiber components. This fiber-integrated approach for nonlinear temporal compression and supercontinuum generation is demonstrated with a resonant electro-optic frequency comb at 1550 nm. We show how to readily achieve pulses shorter than 60 fs at a repetition rate of 20 GHz. The same technique can be applied to picosecond pulses at 10 GHz to demonstrate temporal compression by 9× and achieve 50 fs pulses with a peak power of 5.5 kW. These compressed pulses enable flat supercontinuum generation spanning more than 600 nm after propagation through multi-segment dispersion-tailored anomalous-dispersion highly nonlinear fibers or tantala waveguides. The same 10 GHz source can readily achieve an octave-spanning spectrum for self-referencing in dispersion-engineered silicon nitride waveguides. This simple all-fiber approach to nonlinear spectral broadening fills a critical gap for transforming any narrowband 10–20 GHz frequency comb into a broadband spectrum for a wide range of applications that benefit from the high pulse rate and require access to the individual comb modes.

## I. INTRODUCTION

There is growing interest in broadband, low-noise optical frequency combs at repetition rates in the range of 10–30 GHz for a wide range of applications such as astronomical spectrograph calibration,^1–4^ high-speed precision spectroscopy,^5–9^ low-noise microwave generation,^10–12^ and optical arbitrary waveform generation.^13^ Our work is particularly motivated by the need for simple and robust approaches to 10+ GHz comb generation for astronomical spectrograph calibration. Initial efforts to generate such high repetition rate combs for astronomy relied on filtering a subset of modes from mode-locked lasers (MLLs).^14–19^ However, studies have shown that this approach can lead to calibration errors due to the amplification of unwanted modes through four-wave mixing.^15,20^ As a result, recent studies have directly realized astrocombs with large mode spacings using electro-optic modulation (EOM or EO),^2,21^ microcombs,^3,22^ and cascaded four-wave mixing.^23,24^

Electro-optic modulation of a continuous wave (CW) laser to generate frequency combs with GHz mode spacing has the additional benefits of simplicity, robustness, reliability, and flexibility in independently tuning the operating wavelength as well as the mode spacing.^25–28^ However, generating EO frequency combs with sufficient bandwidth typically requires a cascaded series of phase modulators (PMs) driven by multiple Watts of microwave power. For example, in order to generate a comb with a bandwidth of 10 nm, each cascaded modulator is typically driven by radio frequency signals with average powers in excess of 3 W. As a result, the entire comb generation process is quite costly, and the components require active cooling for long-term operation. A simpler and more cost-effective approach employs the novel technique of resonant EO modulation, as first implemented by Kourogi *et al.*^29^ Utilizing highly efficient resonant EO comb generators, frequency combs with tens of GHz mode spacing and bandwidths of more than 50 nm can be generated using about ten times lower RF power as compared to cascaded EO modulators.^30^ We leverage this comb generation approach using a commercially available package consisting of a fiber-coupled waveguide EO phase modulator within a resonant Fabry–Pérot cavity^31^ to generate frequency combs at 1.55 *μ*m with repetition rates in excess of 10 GHz.

While electro-optic frequency combs and microcombs directly provide mode spacings in the range of tens of GHz, a remaining challenge for these sources lies in matching this large mode spacing with the spectral coverage (hundreds of THz) required, for example, to span the entire bandwidth of an astronomical spectrograph. EOM combs^32,33^ and microcombs^3,34,35^ only give rise to a few hundred femtosecond (fs) pulses in the time domain, whereas traditional mode-locked lasers^36–41^ can directly yield sub-100 fs pulses as required for low noise supercontinuum generation.^42,43^ Despite many efforts in this direction, producing sufficient pulse peak power to initiate nonlinear processes for broadband supercontinuum generation still remains a limitation for bulk as well as chip-integrated frequency combs at tens of GHz repetition rates.^44^ To this end, many previous studies have investigated nonlinear temporal compression from a few hundred femtoseconds (fs) down to tens of fs using three primary approaches. For high-energy pulses in the microjoule or millijoule range with kilohertz to megahertz repetition rates, self-phase modulation (SPM) in gases and solids inside multi-pass cells has been employed, followed by an external grating compressor.^45,46^ Such sources provide gigawatt-to-terawatt peak powers, which places them beyond our regime of interest. On the other hand, for picojoule and nanojoule pulse energies, soliton self-compression has been explored in various media such as fibers,^47–49^ nanophotonic waveguides,^50^ and microresonators.^51^ While simple to implement, soliton self-compression requires a careful balance of dispersion and nonlinearity. Finally, and most relevant to our work, are systems in the pico and nanojoule regimes that employ alternating normal and anomalous dispersion.^52–59^ For instance, self-phase modulation in highly nonlinear fibers (HNLFs) has been employed, where the resulting spectrally broadened pulses are compensated for their chirp using diffraction gratings,^30,33,60,61^ pulse shapers,^62–64^ or anomalous dispersion fibers.^65,66^ However, previous implementations for temporal compression of picojoule pulses fall short in terms of the quality of the compressed pulse, the resultant peak power, or the simplicity of the technique.

To address this challenging and important problem, we introduce a simple and all-PM-fiber temporal compression approach along with its rigorous numerical modeling that defines the complete input parameter space for temporal compression of a broad range of micro-, electro-optic, and semiconductor-based combs. Using this technique, we demonstrate how to generate frequency comb pulses as short as 57 fs with a 20 GHz resonant EO comb generator. Compensation of the nonlinear spectral phase results in pulses with kilowatt peak power and less energy in the pedestals. Using the same technique but with picosecond pulses at 10 GHz, we demonstrate temporal compression by a factor of 9× and achieve pulses as short as 50 fs. Extending our fiber design, we also predict generating a sub-60 fs pulse from the 2.2 ps wide temporal output of a cascaded electro-optic comb. The nonlinear temporal compression is followed by a flat broadband supercontinuum generation in carefully designed multi-segment dispersion-tailored fibers or nanophotonic waveguides. The entire nonlinear system of temporal compression and supercontinuum generation has been modeled, and the simulation results show excellent agreement with the experiment. Our approach to nonlinear spectral generation with off-the-shelf telecom components achieves simplicity, robustness, and long-term stability that can be applied to transform any narrowband frequency combs at these repetition rates into a broadband spectrum.

## II. ALL-FIBER APPROACH FOR TEMPORAL COMPRESSION TO FEMTOSECOND PULSES

**FIG. 1.**
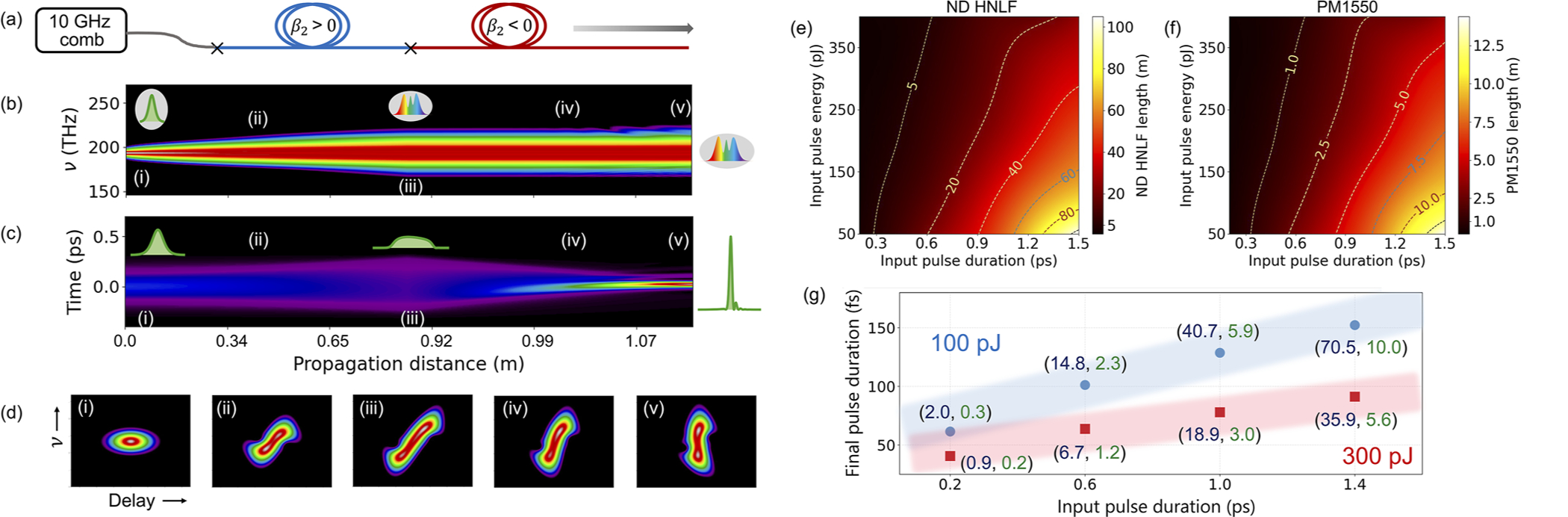
All-fiber approach for nonlinear temporal compression. (a) Schematic of the all-fiber temporal compressor. The 10 GHz comb pulses are amplified and sent through ND HNLF (*β*_2_ > 0) for spectral broadening and then through an appropriate length of PM1550 with anomalous dispersion (*β*_2_ < 0) at 1550 nm to produce sub-50 fs pulses. (b) Simulated spectral evolution of an ideal Gaussian pulse with full width at half maximum (FWHM) pulse duration of 250 fs and pulse energy of 250 pJ propagated through the temporal compression stage consisting of 0.9 m ND HNLF [D = −2.6 ps/(nm km)] and 21 cm of PM1550. (c) Simulated temporal evolution of the 250 pJ, 250 fs input Gaussian pulse through the all-fiber temporal compressor. The compressed pulse is 49 fs. (d) Spectrograms of the pulse as it propagates through the all-fiber temporal compressor. The images correspond to the propagation distances marked in parts (b) and (c). (e) Optimal ND HNLF length (in meters) for temporal compression of a Gaussian pulse with initial duration and energy in the range of 0.2–1.5 ps and 50–400 pJ. (f) Corresponding PM1550 length (in meters) required to compensate for the normal chirp due to the ND HNLF. (g) Pulse durations achievable through simulation of the all-fiber temporal compressor with various input pulse durations and two different pulse energies of 100 and 300 pJ. The parentheses list the required ND HNLF and PM1550 fiber lengths (in meters).

Our all-fiber approach is based on self-phase modulation (SPM) in a polarization-maintaining normal dispersion highly nonlinear fiber (ND HNLF) and the subsequent dispersion compensation in an appropriate length of anomalous dispersion fiber. The main advantage of this technique over soliton self-compression is the lower noise amplification associated with the inhibition of modulation instability in the normal dispersion regime. Figure 1(a) illustrates our approach. The 10 GHz comb pulses are amplified and sent through the ND HNLF for spectral broadening. As a pulse with high peak power (*P*_0_) propagates through HNLF with length *L*, an intensity-dependent nonlinear phase shift is imparted to the pulse. The maximum value of this phase shift is given by *γP*_0_*L* at the peak, where *γ* is the nonlinear coefficient of the fiber. As a result, the pulse acquires a normal chirp, i.e., the leading edge of the pulse is red-shifted in frequency, and the trailing edge is blue-shifted. In the presence of normal group-velocity dispersion (GVD), the red components travel faster than the blue components, leading to additional chirp on the pulse. An analytical expression for spectral broadening becomes more involved with the interplay between SPM and GVD in meter-long fibers and demands numerical analysis. We study the spectral broadening process via numerical modeling of pulse propagation by solving the generalized nonlinear Schrödinger equation (GNLSE) as given below through the split-step Fourier method in PyNLO,^67–69^

(1) $$\begin{array}{l} \frac{\partial E(z,t)}{\partial z}=i\sum_{k\geq 2}\frac{i^{k}\beta_{k}}{k!}\frac{\partial^{k}E}{\partial t^{k}}+i\gamma \left(1+\frac{i}{\omega_{0}}\frac{\partial }{\partial t}\right)\\ \;\times \left[E(z,t)\left(\int_{-\infty }^{t}R\left(t^{\prime }\right){\left|E\left(z,t-t^{\prime }\right)\right|}^{2}dt^{\prime }\right)\right]. \end{array}$$

Here E(z,t) is the complex pulse envelope, $\beta_{k}$ refers to the second and higher orders of fiber dispersion, the second term on the right-hand side of Eq. (1) corresponds to the Kerr nonlinear effects including self-steepening and the last term incorporates Raman effects.

Figures 1(b) and 1(c) show the simulated spectral and temporal evolution of a Gaussian pulse with a full width at half maximum (FWHM) of 250 fs and 250 pJ energy (average power of 2.5 W at 10 GHz) as it propagates through the temporal compression fiber stage comprised of 0.9 m ND HNLF and 21 cm PM1550. Here, we are using the parameters of commercial Optical Fiber Solutions (OFS) ND HNLF with dispersion values of −2.6 ps/(nm km), D_slope_ of 0.026 ps/(nm^2^ km), and nonlinearity parameter of 10.5 W^−1^ km^−1^ at the pump wavelength of 1550 nm. In simulations, the pulse propagates through ND HNLF until the broadened spectrum supports the shortest possible pulse (sub-100 fs). However, longer HNLF adds higher order dispersion terms to the spectral phase of the pulse that cannot be compensated by the quadratic anomalous dispersion of polarization-maintaining 1550 nm fiber (PM1550). Taking into account both of these counteracting effects, the ND HNLF length is fixed at 0.9 m such that the broadened bandwidth supports a short, sub-100 fs pulse while minimizing the higher order spectral phase. The pulse spectrogram at different positions through ND HNLF is shown in Fig. 1(d), which clearly illustrates the normal chirp imparted to the pulse as well as the spectral and temporal broadening.

In order to obtain close to transform-limited pulse duration, the normal chirp has to be compensated. This is usually performed with a grating compressor to provide the required anomalous chirp.^30,49,60^ Here, we use an all-fiber approach in which an appropriate length of PM1550 with anomalous dispersion at 1550 nm replaces the grating compressor. The nonlinearity of PM1550 is quite low compared to HNLF. As a result, further spectral broadening is insignificant, and dispersion compensation is employed to get near transform-limited pulse durations. Figure 1(c) illustrates the temporal evolution of the given input pulse in the all-fiber temporal compressor to give a compressed pulse duration of 49 fs with 4.4 kW peak power. The output pulse spectrogram in Fig. 1(d) shows that most of the normal chirp imparted to the pulse from ND HNLF has been compensated for on propagation through PM1550.

**FIG. 2.**
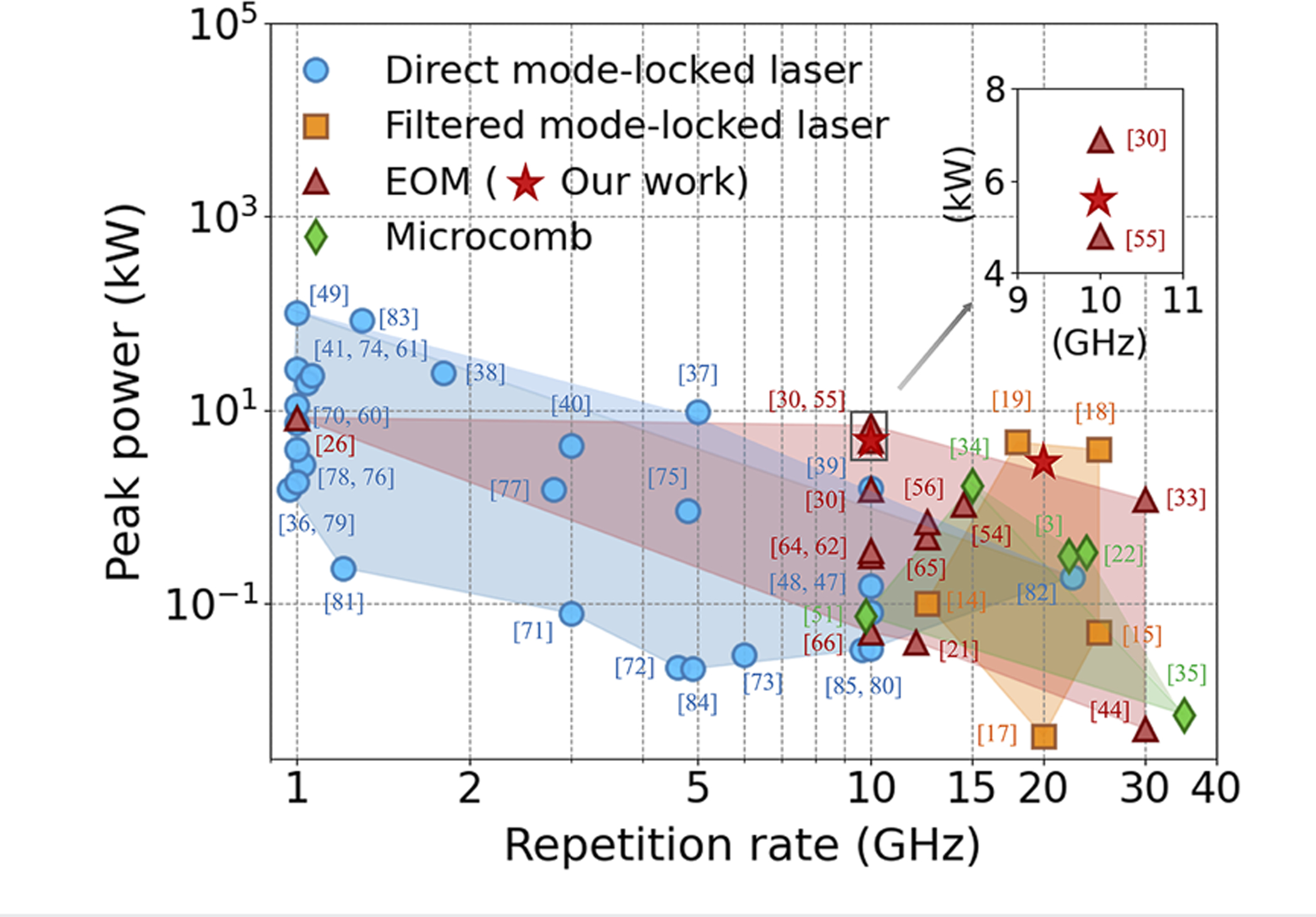
Overview of the peak powers obtained through state-of-the-art nonlinear temporal compression of GHz frequency combs. The inset figure shows a zoomed-in portion of the plot as marked by the box at a 10 GHz repetition rate. The maximum peak powers achieved in this work at repetition rates of 10 and 20 GHz are labeled as red stars.

We generalize this design for a broad range of comb generation approaches by performing simulations to estimate the lengths of fiber required to compress a given input pulse at ~10 GHz repetition rates to sub-150 fs. This all-fiber nonlinear temporal compression technique is independent of the repetition rate of the combs for the given input pulse duration and energy. Figure 1(e) shows the approximate length of ND HNLF, and Fig. 1(f) shows the corresponding PM1550 length required for temporal compression of a given input pulse with a duration ranging from 200 fs to 1.5 ps and energy varying from 50 to 400 pJ. These values correspond to an average power of 0.5–4 W at a 10 GHz repetition rate. Using this combination of ND HNLF and PM1550, Fig. 1(g) provides the achievable compressed pulse duration for the given input pulse characteristics. With this technique, it is possible to compress a 1.4 ps input pulse to 150 fs at pulse energies as low as 100 pJ. Even though this gives an estimate of the achievable compressed pulse duration, for the best prediction of the actual compressed pulse duration, it is necessary for the input pulse in the simulations to resemble the experimentally recorded one. Figure 1(g) also illustrates the applicability of this simple technique to a wide range of input pulse durations, from hundreds of fs to ps, and energies typical of various techniques of GHz comb generation. As we show below, this all-fiber nonlinear temporal compression approach has been applied to a resonant electro-optic comb generator (REOCG) at microwave frequencies of 10 and 20 GHz to obtain pulse durations between 50 and 60 fs. The peak powers of the compressed pulses achieved in this work, labeled as red stars in Fig. 2, are compared to other state-of-the-art nonlinear temporal compression approaches for GHz repetition rates.^70–85^ A few important features differentiate our work from the adjacent values in Fig. 2. For example, the average power at 20 GHz repetition rate in our work is ~ 4× lower than that of Refs. 18 and 19. Those works employed more complicated modefiltering cavities and amplification to average powers of 12–15 W to get compressed pulse durations of 130–140 fs. In another case, the peak power of ~6.9 kW inferred from Ref. 30 involves a second stage of supercontinuum generation in a silicon nitride waveguide and the subsequent dispersion compensation using silica glass in free space, in addition to the initial stage of spectral broadening in highly nonlinear fiber and temporal compression using diffraction gratings that provided 1.5 kW of peak power. Most similar to our work is that of Obrzud,^55^ where lengths of fiber with alternating normal and anomalous dispersion were employed. However, that work did not provide details on the modeling or fiber lengths employed.

## III. DEMONSTRATION IN A RESONANT ELECTRO-OPTIC COMB

### A. All-fiber temporal compression to sub-60 fs

**FIG. 3.**
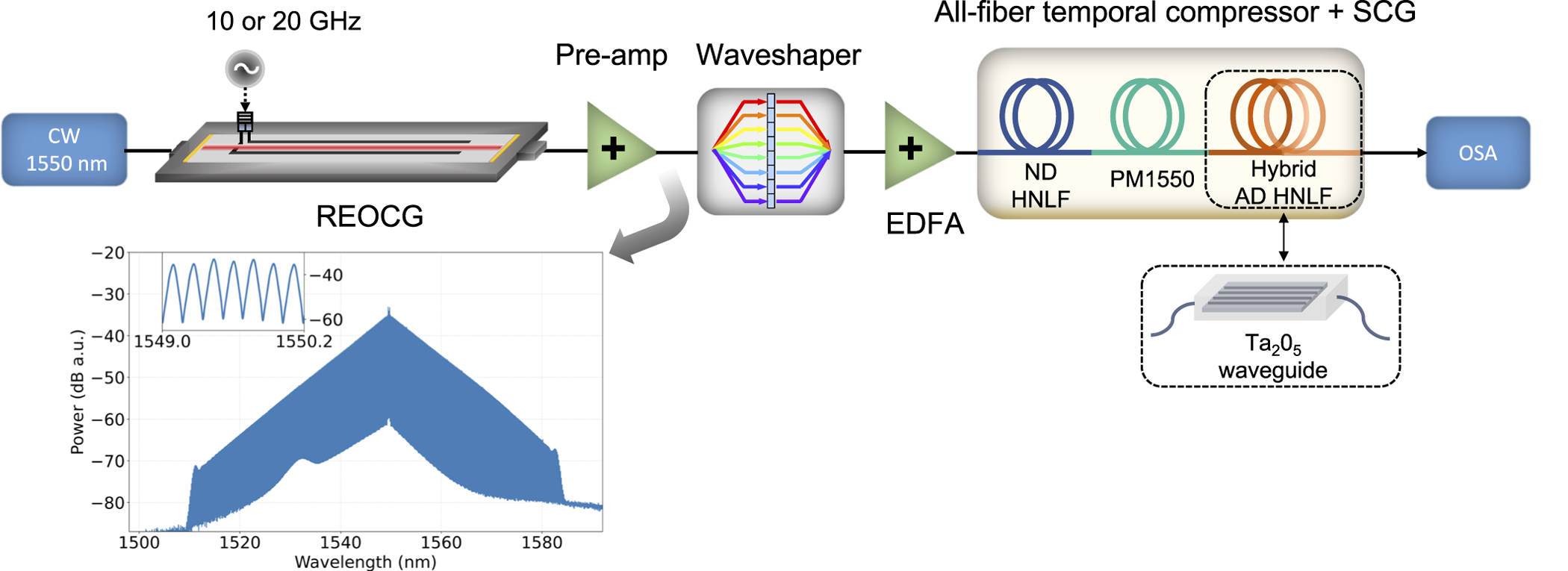
Experimental setup for nonlinear temporal compression and supercontinuum generation of the resonant electro-optic frequency comb. The blue trace shows the output of the 20 GHz comb generator, and the inset shows the 20 GHz comb modes. The compressed pulses were sent to anomalous dispersion highly nonlinear fibers or nanophotonic waveguides like tantala and silicon nitride for supercontinuum generation. REOCG: resonant electro-optic comb generator, EDFA: erbium-doped fiber amplifier, ND HNLF: normal dispersion highly nonlinear fiber, PM1550: polarization-maintaining single-mode fiber at 1550 nm, AD HNLF: anomalous dispersion highly nonlinear fiber, Ta_2_O_5_: tantalum pentoxide, SCG: supercontinuum generator, OSA: optical spectrum analyzer.

**FIG. 4.**
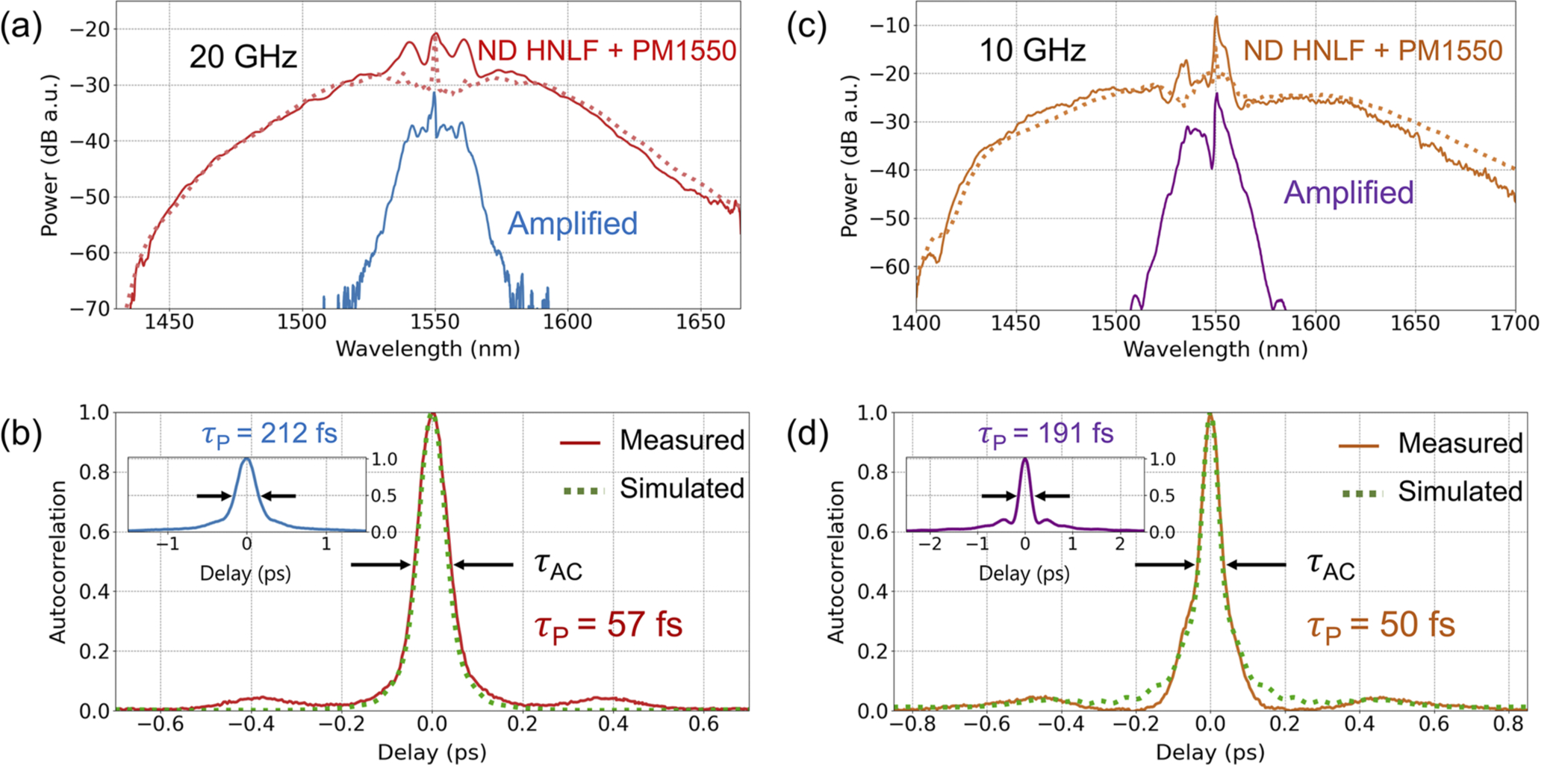
(a) Optical spectra recorded after amplification of the 20 GHz comb pulses to 3 W (blue trace) and propagation through a temporal compression stage consisting of 1.2 m of ND HNLF and 22 cm of PM1550 (solid red trace). The dotted red trace gives the simulated spectrum. The traces are offset by 10 dB for clarity. (b) Measured autocorrelation after amplification (blue trace) and temporal compression (red trace) of the 20 GHz comb. The dotted green trace shows the calculated autocorrelation of the compressed pulse obtained from the NLSE simulation. The pulse duration $\left(\tau_{P}\right)$ is calculated from the autocorrelation width $\left(\tau_{AC}\right)$, considering a deconvolution factor of 1.41. The measured FWHM of the compressed pulse (57 fs) is very close to the simulated value of 52 fs (dotted green trace). (c) Optical spectra after amplification of the 10 GHz comb pulses (purple trace) and propagation through the same fiber temporal compressor (orange trace). The dotted orange trace gives the simulated spectrum. The traces are offset by 15 dB for clarity. (d) Corresponding autocorrelation traces recorded for the 10 GHz comb. The measured FWHM of the compressed pulse at 10 GHz is 50 fs, while the simulation (dotted green trace) gives 45 fs.

Our electro-optic comb generation employs a fiber-integrated waveguide phase modulator in a resonant Fabry–Pérot cavity (model: WTEC-01).^31^ The so-called resonant electro-optic comb generator (REOCG) has the advantages of highly efficient resonance modulation and built-in RF phase noise filtering.^86^ Figure 3 shows the schematic of our experimental setup. A cavity-stabilized continuous wave laser at 1550 nm with ~25 mW power is used to seed the comb generator. The 1550 nm laser employed here is a fiber laser stabilized in a high-Finesse cavity and has a 1 s Allan deviation (Adev) of ~3 × 10^−15^. A dielectric resonant oscillator (DRO Synergy model: KDFLOD1000–8) phase-locked to a 10 MHz maser signal is frequency doubled to 20 GHz and then amplified to an average power of 1 W. The REOCG is driven by this 1 W microwave signal at 20 GHz that is a multiple of the Fabry–Pérot cavity’s free spectral range (FSR = 2.5 GHz). The optical spectrum of the resonant EOM comb has a double-sided exponentially decaying shape, as shown in Fig. 3. More details about the REOCG can be found in the supplementary material. The comb pulses are sent through a programmable pulse shaper (Finisar 1000S) for dispersion compensation and to apply the required group delay across one side of the spectrum in order to overlap the two interleaved pulse trains while maintaining the entire optical system in fiber-coupled components. The pulse shaper is not critical for achieving close to transform-limited pulses since it can be replaced by an appropriate length of single-mode fiber for nearly quadratic dispersion compensation. The 20 GHz pulse train is then amplified to 3 W average power using an erbium-doped fiber amplifier (EDFA). The intensity autocorrelation width of the amplified pulses after nonlinear broadening in erbium-gain fiber is measured to be ~300 fs [inset in Fig. 4(b)]. Assuming a Gaussian pulse shape with a deconvolution factor of 1.41, the resultant pulse duration is 212 fs, which is close to the band-limited pulse duration of 195 fs. This deconvolution factor has been verified in all cases by calculating the autocorrelation pulse duration from the band-limited pulse.

Although the REOCG generates pulses as short as 212 fs after amplification, previous studies have shown that pulse durations greater than 100 fs adversely affect the bandwidth as well as the coherence of the supercontinuum generated in anomalous dispersion fibers.^42,43^ In order to overcome this issue, we compress this pulse to less than 60 fs using an all-fiber approach, as described in the previous section. Figure 1(g) shows that ~ 2 m of normal-dispersion (ND) highly nonlinear fiber (HNLF) broadens an ideal Gaussian pulse of 200 fs and 100 pJ to a spectral bandwidth that supports a 50 fs pulse. Assuming an average power of 2.95 W (pulse energy of 148 pJ at 20 GHz), simulation results show that 1.2 m of ND HNLF is required for the first stage of spectral broadening through self-phase modulation (SPM), as depicted by the dotted red trace in Fig. 4(a). To maintain close agreement with the experiment, we anomalously chirp the Fourier transform-limited pulse of the amplified 20 GHz comb spectrum and use it as input to the simulations. On propagating the spectrally broadened pulse through 22 cm of PM1550 to overcome the normal chirp, modeling predicts a compressed pulse duration of 52 fs [Fig. 4(b)]. Experimentally, we were able to measure a broadened spectrum [solid red trace in Fig. 4(a)] and compressed pulse with a full width at half maximum (FWHM) of 57 fs after propagating the amplified pulses through 1.2 m of ND HNLF and 22 cm of PM1550, in exact agreement with the model. Figure 4(b) shows the autocorrelation traces at the output of PM1550 in our setup (red solid curve) as well as through simulations (dotted green curve) and again demonstrates excellent agreement between the two. The average output power from the PM1550 fiber is measured to be 2.8 W. This results in a 57 fs pulse with a peak power of ~2.4 kW.

In order to demonstrate the robustness of our temporal compression scheme in tunable comb systems, we changed the repetition rate of our resonant electro-optic comb to 10 GHz and employed the same setup as illustrated in Fig. 3. Here, the pulse shaper is used to remove half of the optical spectrum to generate a single 10 GHz pulse train (more information is provided in the supplementary material). The 10 GHz pulses are amplified to 3 W using an EDFA, and the resultant intensity autocorrelation pulse duration is measured to be 270 fs, which corresponds to a pulse duration of 191 fs, assuming a deconvolution factor of 1.41 [inset in Fig. 4(d)]. On propagating these amplified pulses through the same fiber compression stage consisting of 1.2 m long ND HNLF [D = −2.6 ps/(nm km)] and 22 cm PM1550, the pulse duration is reduced to 50 fs. The corresponding measured autocorrelation (orange trace) shows good agreement with the simulated result [dotted green trace in Fig. 4(d)]. The Fourier transform-limited pulse duration of the spectrum recorded after the compression fiber stage is ~30 fs. The compressed pulse is slightly chirped as opposed to the 20 GHz case since the fiber compressor design is not optimized for the 10 GHz system.

### B. Supercontinuum generation in anomalous dispersion HNLF and SiN waveguide

**FIG. 5.**
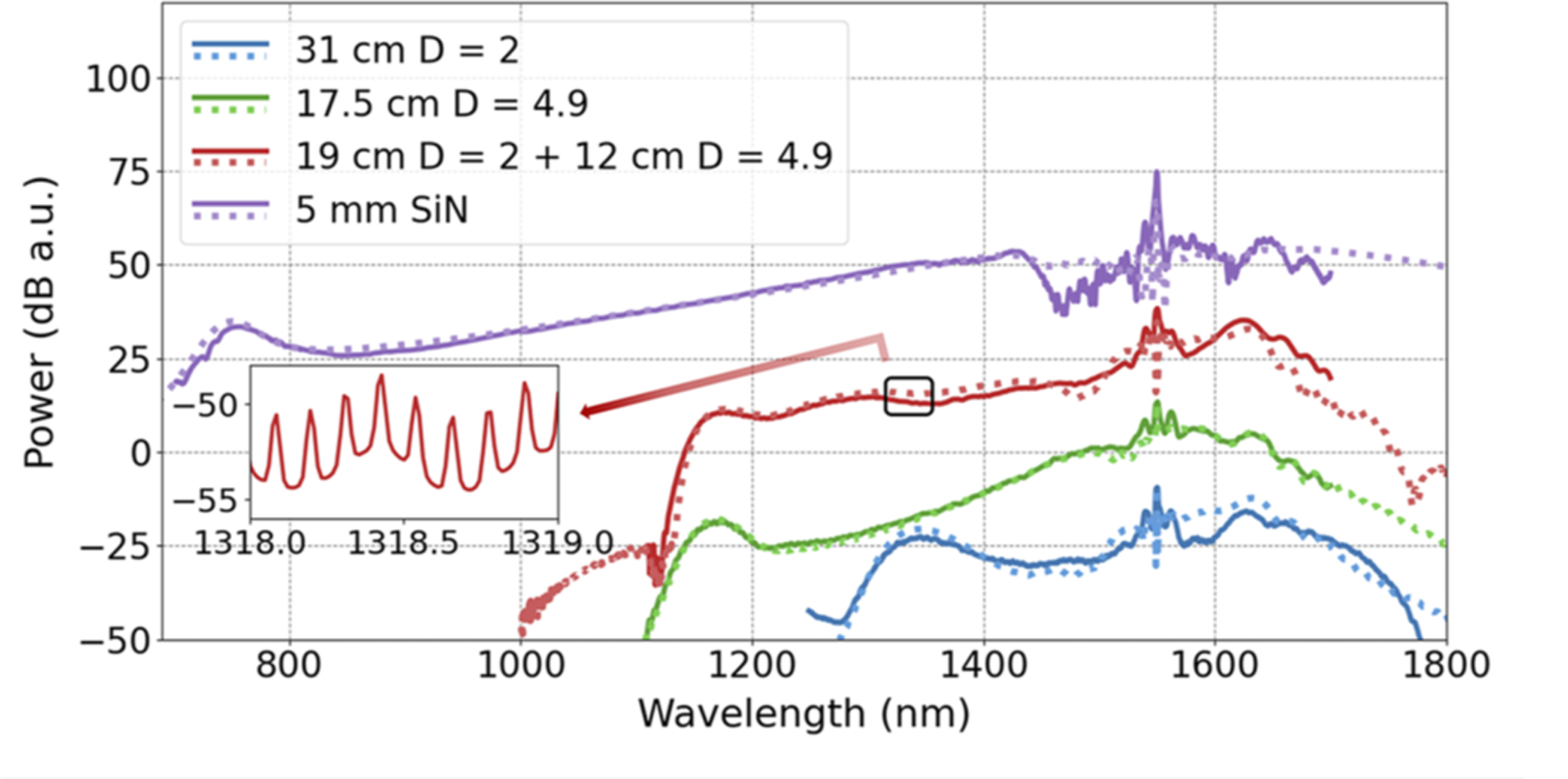
Supercontinuum spectra generated with the compressed pulses shown in Fig. 4. The blue, green, and red traces are from the 57 fs driving pulse at 20 GHz in different lengths of AD HNLF with dispersion values of D = 2 ps/nm km (blue), D = 4.9 ps/nm km (green), and hybrid (red). The purple trace corresponds to an octave-spanning supercontinuum obtained in a 5 mm long SiN waveguide from the 50 fs driving pulse at 10 GHz. Experimental and simulation results are shown as solid and dotted lines, respectively. The inset shows the 20 GHz comb modes around 1319 nm of the supercontinuum generated from hybrid AD HNLF. The blue, green, and red traces are vertically offset by 20 dB, and the purple trace is offset by 30 dB for clarity.

The compressed pulse at 20 GHz is then passed through anomalous dispersion (AD) HNLF for supercontinuum generation. We studied the supercontinuum generated in two AD HNLFs with different dispersion characteristics independently and observed that a flat broadband spectrum is generated on propagation through multi-segment AD HNLF at the point of soliton fission. Figure 5 shows the corresponding spectra obtained experimentally (solid curves) as well as through modeling (dotted curves). The blue trace corresponds to the supercontinuum generated using 31 cm long HNLF with lower anomalous dispersion at the pump wavelength [(D = 2.2 ps/(nm km) and D_slope_ = 0.026 ps/(nm^2^ km)]. The green trace indicates the supercontinuum obtained in 17.5 cm long HNLF with higher AD [D = 5.4 ps/(nm km), and D_slope_ = 0.028 ps/(nm^2^ km)]. The HNLF with lower AD generates a dispersive wave (DW) around 1345 nm (blue trace), whereas the one with higher AD can produce DW at 1156 nm, wavelengths further away from the pump (green trace). The total splicing loss through the combined temporal compression and supercontinuum generation fiber system is measured to be ~1.5 dB. This loss is attributed to the splicing between PM1550 and the smaller core HNLF and has been included in the input pulse energy to the respective fibers in the simulation. Since the dispersion parameters of HNLF given by the manufacturer are only valid over a small wavelength band of ~100 nm around 1550 nm, the fiber dispersion parameters in simulations were tuned to match the location of the dispersive wave in the broad supercontinuum observed experimentally. This tuning is performed after initially fixing the input pulse duration and energy to the measured values. The corresponding dispersion parameters used in simulations are D = 2 ps/(nm km), D_slope_ = 0.034 ps/(nm^2^ km), and D = 4.9 ps/(nm km), D_slope_ = 0.023 ps/(nm^2^ km). This approach gives an efficient way to determine the dispersion parameters of the commercially available HNLF over a broad spectral range.

A smooth supercontinuum spanning 1150–1700 nm (red trace in Fig. 5) is generated in a hybrid HNLF consisting of 19 cm long D = 2 ps/(nm km) and 12 cm long D = 4.9 ps/(nm km). The length of the first lower dispersion AD HNLF is fixed before the point of soliton fission, and then the pulse train is propagated through an appropriate length of the second AD HNLF to obtain a flat broader spectrum. The zoomed-in trace of the resolved 20 GHz comb modes of the supercontinuum at 1319 nm measured at 0.02 nm resolution on an optical spectrum analyzer is shown in the inset of Fig. 5. The total average power measured in free space after the multi-segment AD HNLF is ~2.5 W, which corresponds to more than 20 *μ*W/mode around the dispersive wave at 1150 nm.

In order to use these broadband spectra for high-impact metrology applications, we need a self-referenced frequency comb, which in turn requires an octave-spanning spectrum. We show that the temporally compressed 50 fs, 10 GHz comb source developed in this work can readily generate an octave-spanning spectrum (purple trace in Fig. 5) in a dispersion-engineered silicon nitride (SiN) waveguide. The waveguide fabricated by the open access foundry Ligentec is 5 mm long with a cross-section of 800 × 2500 nm^2^ and is silica clad. The average power incident on the waveguide is 2.8 W, and the measured total insertion loss is ~4.3 dB. Considering this reduction in input pulse energy as well as the calculated frequency-dependent effective index and nonlinearity parameter of the waveguide via the finite difference mode solver, the simulated spectrum (dotted purple trace in Fig. 5) closely agrees with the recorded one.

### C. Extending this approach to picosecond input pulses

**FIG. 6.**
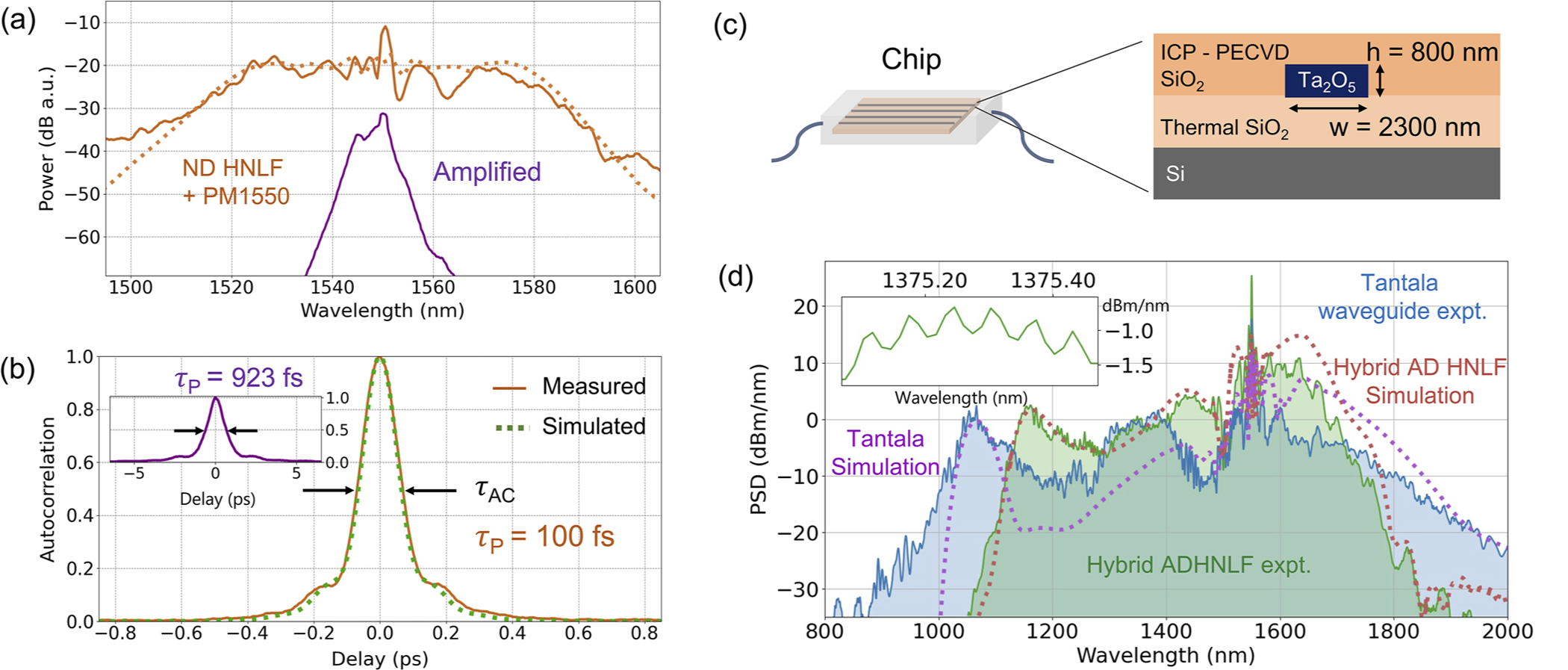
(a) Optical spectra after amplification of the spectrally compressed, 920 fs 10 GHz comb pulses to 3 W (purple trace) and propagation through a temporal compression stage consisting of 8.4 m of ND HNLF and 2 m of PM1550 (orange trace). The dotted orange trace shows the simulated spectrum. The traces are offset by 18 dB for clarity. (b) Measured autocorrelation after amplification (purple trace) and temporal compression (orange trace) of the 10 GHz comb. The measured pulse duration ($\tau_{P}=100$ fs) is calculated from the autocorrelation width ($\tau_{AC}$), considering a deconvolution factor of 1.41. The dotted green trace shows the calculated autocorrelation of the compressed pulse (duration of 93 fs) obtained from the NLSE simulation. (c) Fiber-connectorized tantala (Ta_2_O_5_) chip and its waveguide cross-section. (d) Supercontinuum spectra generated from the compressed 100 fs 10 GHz pulses in a multi-segment AD HNLF (green trace) consisting of 23 cm of D = 2 ps/(nm km) and 8 cm of D = 5.6 ps/(nm km) and in the tantala waveguide (blue trace). The dotted traces represent simulation results in the multi-segment AD HNLF (red) and tantala waveguide (purple). The inset figure shows the 10 GHz comb modes around 1375 nm of the supercontinuum obtained using the hybrid AD HNLF.

Since electro-optic comb generators generally give picosecond pulses in the time domain, we designed and implemented our all-PM fiber temporal compression technique for longer picosecond input pulses. Initially, the spectral bandwidth of our 10 GHz comb is reduced using the pulse shaper such that the intensity autocorrelation after amplification to 3 W gives a 923 fs pulse on deconvolution [inset in Fig. 6(b)]. Then, we propagate the amplified pulses through 8.4 m of ND HNLF [D = −2.6 ps/(nm km)] and 2 m of PM1550 to achieve a compressed pulse duration of 100 fs, as shown by the orange autocorrelation trace in Fig. 6(b). Assuming chirped transform-limited pulses of the amplified comb spectrum as the input, modeling results on the propagation of the amplified pulses through 8.4 m of ND HNLF and 1.82 m of PM1550 give a compressed pulse duration of 93 fs, which is in close agreement with the experiment.

The resulting 100 fs pulse is then sent through a different multi-segment dispersion-tailored AD HNLF for supercontinuum generation. Figure 6(d) shows the supercontinuum (blue curve) generated in hybrid AD HNLF consisting of 23 cm long D = 2 ps/(nm km) fiber and 8 cm long D = 5.6 ps/(nm km) fiber, and the corresponding modeled spectrum is depicted in the red dotted trace. These fiber dispersion parameters and the nonlinearity parameter value of 10.5 W^−1^ km^−1^ are given by OFS. The simulation results demonstrate better agreement with the experiment on tuning the HNLF dispersion parameters to be D = 1.3 ps/(nm km), D_slope_ = 0.03 ps/(nm^2^ km), and D = 5.8 ps/(nm km), D_slope_ = 0.027 ps/(nm^2^ km), respectively, for the given input pulse. A broad flat spectrum from 1160 to 1660 nm, as shown in the green trace of Fig. 6(d), is obtained at the end of the multi-segment fiber stage. The 10 GHz comb modes around 1375 nm are depicted in the inset of Fig. 6(d).

Finally, we demonstrate a 10 GHz supercontinuum generated from this 100 fs pulse in a specially designed fiber-packaged tantalum pentoxide (Ta_2_O_5_) or tantala waveguide module fabricated by Octave Photonics. The tantala waveguide is 19 mm long with a cross-section of 800 × 2300 nm^2^ [Fig. 6(c)]. The waveguides have silica cladding and are fabricated on a silicon wafer. The average power incident on the waveguide is ~1.7 W, and the total insertion loss is measured to be 5.4 dB. We observed a flat supercontinuum extending from 1050 to 1700 nm in wavelength [blue trace in Fig. 6(d)]. The simulation result in the tantala waveguide given by the purple dotted trace in Fig. 6(d) shows reasonable agreement with the experimentally measured spectrum. We estimated the nonlinearity parameter of Ta_2_O_5_ at 1550 nm to be 1.2 W^−1^ m^−1^ and calculated the frequency-dependent effective index of the waveguide via the finite difference fully vectorial mode solver in our nonlinear pulse modeling. The difference in the supercontinuum bandwidth obtained from the tantalum waveguide as compared to the SiN waveguide in Sec. III B can be attributed to the difference in waveguide dispersion, nonlinearity, as well as input pulse duration. The SC spectra appear to have more complicated intensity modulations as compared to the smooth spectra in Fig. 5 because the fundamental solitons have broken apart from the main pulse at the end of pulse propagation.

## IV. CONCLUSIONS AND FUTURE WORK

We presented a simple, all-PM-fiber approach for efficient nonlinear temporal compression to femtosecond pulses and supercontinuum generation from 10+ GHz frequency combs. Using this scheme, we generated ultrashort pulses of duration 50 and 57 fs with peak powers of 5.5 and 2.4 kW, respectively, at 10 and 20 GHz repetition rates. These pulses originated from a resonant fiber-integrated waveguide-type electro-optic comb generator. We have also demonstrated a flat supercontinuum spanning over 500 nm with more than 20 *μ*W/mode from these compressed pulses in a multi-segment dispersion-tailored AD HNLF. The compressed pulses enabled the generation of an octave-spanning spectrum in a SiN waveguide, which in turn showed the potential to stabilize our 10 GHz comb through self-referencing. By employing this pulse compression scheme, we achieved a high temporal compression ratio of 9× for picosecond input pulses at 10 GHz and generated a supercontinuum that spans 1050–1700 nm in tantala waveguides. The simulation results on nonlinear pulse propagation have shown excellent agreement with the experiment throughout.

The simplicity and robustness of our nonlinear spectral broadening technique with off-the-shelf telecom components make it compatible with other techniques of comb generation and well-suited for applications such as astronomical spectrograph calibration and the fast acquisition of precise spectroscopic data. Our 10+ GHz comb system also shows promise for generating visible astrocombs via sum frequency generation in periodically poled lithium niobate waveguides^87^ and mid-infrared combs for high-speed spectroscopy via intra-pulse difference frequency generation in nonlinear crystals.^49,88^ Future work will include the study of the coherence and relative intensity source (RIN) of the supercontinuum, with a focus on understanding the effect of the intrinsic electro-optic cavity filtering on broadband microwave thermal noise.

## Supplementary Material

Supp1

See the supplementary material for more information on REOCG, heterodyne beat measurement, and temporal compression of cascaded EO combs.

## Data Availability

The data that support the findings of this study are available from the corresponding author upon reasonable request.
